# Differential *Salmonella* Typhimurium intracellular replication and host cell responses in caecal and ileal organoids derived from chicken

**DOI:** 10.1186/s13567-023-01189-3

**Published:** 2023-07-31

**Authors:** Sonia Lacroix-Lamandé, Ophélie Bernardi, Tiffany Pezier, Emilie Barilleau, Julien Burlaud-Gaillard, Anissa Gagneux, Philippe Velge, Agnès Wiedemann

**Affiliations:** 1 INRAE, Université de Tours, ISP, 37380 Nouzilly, France; 2 Plateforme IBiSA de Microscopie Électronique, Université de Tours et CHRU de Tours, Tours, France; 3 Present Address: IRSD, Institut de Recherche en Santé Digestive, ENVT, INRAE, INSERM, Université́ de Toulouse, UPS, Toulouse, France

**Keywords:** *Salmonella*, chicken, intestinal segment organoid, bacterial colonization, epithelial cell responses

## Abstract

Chicken infection with *Salmonella* Typhimurium is an important source of foodborne human diseases. *Salmonella* colonizes the avian intestinal tract and more particularly the caecum, without causing symptoms. This thus poses a challenge for the prevention of foodborne transmission. Until now, studies on the interaction of *Salmonella* with the avian gut intestine have been limited by the absence of in vitro intestinal culture models. Here, we established intestinal crypt‐derived chicken organoids to better decipher the impact of *Salmonella* intracellular replication on avian intestinal epithelium. Using a 3D organoid model, we observed a significantly higher replication rate of the intracellular bacteria in caecal organoids than in ileal organoids. Our model thus recreates intracellular environment, allowing *Salmonella* replication of avian epithelium according to the intestinal segment. Moreover, an inhibition of the cellular proliferation was observed in infected ileal and caecal organoids compared to uninfected organoids. This appears with a higher effect in ileal organoids, as well as a higher cytokine and signaling molecule response in infected ileal organoids at 3 h post-infection (hpi) than in caecal organoids that could explain the lower replication rate of *Salmonella* observed later at 24 hpi. To conclude, this study demonstrates that the 3D organoid is a model allowing to decipher the intracellular impact of *Salmonella* on the intestinal epithelium cell response and illustrates the importance of the gut segment used to purify stem cells and derive organoids to specifically study epithelial cell -*Salmonella* interaction.

**Supplementary Information:**

The online version contains supplementary material available at 10.1186/s13567-023-01189-3.

## Introduction

*Salmonella* is an intracellular bacterium, causing important public health and economic problems throughout the world. Salmonellosis is after campylobacteriosis, the second commonest reported zoonosis and food poisoning in Europe, resulting in hospitalization and death [1]. Non‐typhoidal *Salmonella* are the most frequent pathogen isolated in foodborne outbreaks. Human contamination mainly occurs through consumption of poultry products, especially eggs and meat. Contaminated food with *Salmonella* can thus have a negative impact on agrifood industry and trade sectors due to costly recalls of products and by limiting market access. It thus results in significant economic losses and represents a substantial burden on healthcare system.

*Salmonella* can induce several diseases ranging from gastro‐enteritis to typhoid fever according to the host and serotype. In humans, *S*. Enteritidis and *S*. Typhimurium are the two main serotypes responsible for gastro-enteritis. Poultry infections with those serotypes are very insidious as infected animals are usually symptomless carriers. The absence of symptoms makes the identification of infected animals difficult, leading to the production of contaminated poultry meat and eggs that will be consumed by humans.

After oral contamination, the initiating step for *Salmonella* infection requires the interaction of bacteria with the intestinal epithelium. In order to survive in the host cellular environment, *Salmonella* has evolved mechanisms to manipulate host cell functions for its own benefit. *Salmonella* is able to attach to, enter non-phagocytic cells by using three known invasion factors: a type three secretion system (T3SS-1) and 2 invasins (Rck and PagN). It multiplies inside host intestinal cells by following a common pathway leading to the formation of a *Salmonella* containing vacuole (SCV) [2]. Both wild-type *Salmonella* and T3SS1-invalidated *Salmonella* show the maturation of the SCV continued through an acidified phase, leading to *Salmonella* replication [3] and inducing host cellular responses, which are known to be critical for bacterial survival, colonization and establishment of the disease. The presence of *S.* Typhimurium according to the intestinal segment has been studied and pointed out that the caecum is the intestinal segment most highly colonized and also the site of persistence in infected chicks [4, 5]. However, the lack of avian chicken cell lines leads to very little knowledge about the intracellular fate of *S.* Typhimurium, crucial steps allowing the chicken intestinal epithelium colonization.

Until now, avian gut infection by *Salmonella* has only been studied in in vivo models but the recent opportunity to study the bacterial interaction with epithelial cells in organoids [6] offers a unique chance to improve our knowledge. Indeed, isolated primary intestinal epithelial cells in 3D culture reconstitute the crypt architecture (central lumen with surrounding epithelial monolayer) and the cell lineage diversity thanks to the stem cell properties. The first chicken intestinal organoids were obtained from the entire small intestine of chicken embryo [7, 8]. They were cultivated in 3D in Matrigel™ matrix and exposed to a probiotic, a TLR2 ligand [9] or various chemicals [10] to demonstrate a functional epithelium. Many studies have described optimization in the culture conditions with different concentrations of EGF, R-Spondin and of Noggin to improve enrichments, passages and cryopreservation of chicken organoids [11–13]. Two novel methods of cultivation of intestinal chicken organoids were described in order to allow access to the apical side of the cells: the two-dimensional (2D) polarized models of intestinal monolayers and the inside-out organoids [14–16]. Infections by *S.* Typhimurium have been successfully performed in the chicken inside-out enteroids, leading to intracellular bacterial replication but only for 8 h. Despite the different chicken intestinal organoid models described, no study has compared the capacity of *Salmonella* to intracellularly replicate according to intestinal segment and evaluated the cellular response of chicken intestinal epithelium to intracellular *Salmonella*.

The aim of this study was to (1) establish a 3D avian minigut model from ileum and caecum of young chickens, (2) confirm the capacity of this model to recreate intracellular environment, allowing *Salmonella* replication of avian epithelium and (3) investigate the impact of intracellular *Salmonella* on the intestinal cell response.

## Materials and methods

### L-Wnt3a, R-spondin and Noggin (L-WRN) production and medium

L-WRN cell line (ATCC^®^: CRL-3276, LGC Standard, Molsheim, France) secreting Wnt-3A, R-spondin 3, and Noggin was cultured in DMEM (Dulbecco’s modified Eagle’s medium, Gibco Life Technologies, Paisley, UK) containing 4.5 g/L glucose supplemented with 10% fetal bovine serum (FBS; Sigma Aldrich, Saint-Louis, MO, USA), 0.5 mg/mL geneticin (G-418; Sigma Aldrich, Saint-Louis, MO, USA) and 0.5 mg/mL hygromycin (Thermo Fisher Scientific, Illkirch, France) at 37 °C in a humidified atmosphere at 5% CO_2_. After 3 days, cells were washed with DMEM and then further cultured in DMEM containing 4.5 g/L glucose supplemented with 10% FBS. The L-WRN cell supernatant constitutes the L-WRN conditioned medium (L-WRN CM) and was collected every 2–3 days over 12 days. After centrifugation at 300 x *g* for 5 min at room temperature, L-WRN CM was aliquoted and stored at − 20 °C until use. L-WRN cells were weekly sub-cultured (dilution 1:10 v/v) by partial digestion with EDTA-trypsin 0.25% w/v (Thermo Fisher Scientific, Illkirch, France).

### Isolation of intestinal crypts

White Leghorn PA12 chickens from INRAE experimental unit PFIE (Centre Val de Loire, France) were used for intestinal crypt isolation in strict accordance with French legislation and approved by the French Ministry of education, higher education and research (Ministère de l’Education Nationale, de l’Enseignement Supérieur et de la Recherche). Sections of ileal and caecal segments were removed from 4 days old chicken and placed in phosphate-buffered saline (PBS) without Ca^2+^ and Mg^2+^ (ThermoFisher Scientific, Illkirch, France) supplemented with 100 U/mL penicillin and 100 mg/mL streptomycin. Intestinal crypts were isolated according to established protocols [17]. Briefly, tissue was longitudinally opened and cut before being placed in dissociation buffer (PBS without Ca^2+^ and Mg^2+^ containing 9 mM EDTA (Sigma Aldrich, Saint-Louis, MO, USA), 3 mM 1,4-Dithiothréitol (DTT, Sigma Aldrich, Saint-Louis, MO, USA) and 10 µM Y27632 (Tocris, Bristol, UK) in order to dissociate crypts for 45 min with 16 rpm shaking at room temperature. Then, the intestinal fragments were transferred in cold PBS without Ca2^+^ and Mg^2+^ following by 2 min manual vigorous up and down shaking. To remove the intestinal villus, the supernatant was filtered first with a 100 µm cell strainer and then rinsed with PBS without Ca^2+^ and Mg^2+^. This step was repeated with a 70 µm cell strainer and the final filtrate was centrifugated at 220 x *g* for 5 min at room temperature. The pellet containing intestinal crypts was resuspended in DMEM/F12-Glutamax-HEPES (Gibco Life Technologies, Paisley, UK), 5% FBS, 100 U/mL penicillin and 100 mg/mL streptomycin. The number of crypts was estimated using a bright field optical microscopy (Nikon, Champigny-sur-Marne, France). About 2500 crypts were embedded in 50 μL Matrigel™ (Corning, Amsterdam, The Netherlands) mixed at 50% with L-WRN complete medium (DMEM/F-12 Glutamax-Hepes (Gibco Life Technologies, Paisley, UK) supplemented with 50% L-WRN CM, 10 mM HEPES (Gibco Life Technologies, Paisley, UK), B27 1X (ThermoFisher Scientific, Illkirch, France), 50 ng/mL EGF (Sigma Aldrich, Saint-Louis, MO, USA), 500 nM A83-01 (Tocris, Bristol, UK), 10 µM SB2022190 (Tocris, Bristol, UK), 10 nM gastrin I (Tocris, Bristol, UK), 1 mM *N*-acetyl-L-cysteine (Sigma, Saint-Louis, MO, USA), 10 µM SB431542 (Tocris, Bristol, UK) and 10 µM Y27632 (Tocris, Bristol, UK), and seeded in pre-warmed 24-well plates. After Matrigel™ polymerization (about 20 min at 37 °C in a humidified atmosphere at 5% CO_2_), 500 μL L-WRN complete medium/well were added. L-WRN complete medium was changed every 2–3 days.

### Organoid culture and passaging

After 5–8 days of culture, the number of organoids per well was estimated using a bright field optical microscopy (Nikon, Champigny-sur-Marne, France). For weekly passaging at 1:4 to 1:8 split ratios, organoids were washed with warm PBS without Ca^2+^ and Mg^2+^ and then, enzymatically dissociated using TrypLE™ (Gibco Life Technologies, Paisley, UK) according to manufacturer recommendations. Depending on the number of organoids and the splitting ratio, the corresponding quantity of dissociated organoid was then embedded in 50 µL fresh Matrigel™ mixed at 50% with L-WRN complete medium, seeded per well of a 24-well plate and cultured with L-WRN complete medium as described above.

### Transmission electron microscopy (TEM)

Organoids were collected and fixed in 4% paraformaldehyde (PFA), 1% glutaraldehyde (Sigma Aldrich, Saint-Louis, MO, USA) and 0.1 M phosphate buffer (pH 7.2) for 24 h. Samples were then washed in PBS and incubated with 2% osmium tetroxide (Agar Scientific, Stansted, Essex, UK) for 1 h. Organoids were then fully dehydrated in a graded series of ethanol solutions and propylene oxide. The impregnation step was performed with a mixture of (1:1) propylene oxide/Epon resin (Sigma Aldrich, Saint-Louis, MO, USA) and then left overnight in pure resin. Cells were then embedded in Epon resin (Sigma Aldrich, Saint-Louis, MO, USA), which was allowed to polymerize for 48 h at 60 °C. Ultra-thin section (90 nm) of these blocks were obtained with a Leica EM UC7 ultramicrotome (Leica Microsystems, Wetzlar, Germany). Sections were stained with 5% uranyl acetate (Agar Scientific, Stansted, Essex, UK), 5% lead citrate (Sigma Aldrich, Saint-Louis, MO, USA) and observations were made with a transmission electron microscope (JEOL 1011, Tokyo, Japan).

### Gene expression analyses

Total RNA was extracted from about 300 organoids cultivated in 24 well plate after washing with PBS without Ca2^+^ and Mg^2+^ at 37 °C and resuspension in 300 µL Tri-Reagent (Sigma Aldrich, Saint-Louis, MO, USA). The samples were kept at − 80 °C until RNA extraction. After thawing, RNA was extracted with DirectZol miniprep (Zymo Research, Irvine, USA) according to manufacturer’s recommendations. RNA concentration was determined using a Nanodrop (ThermoFisher Scientific, Illkirch, France) before storage at − 80 °C. RT reactions were performed with iScript RT Supermix (Biorad, Hercules, CA, USA) according to manufacturer’s recommandations. Gene expression measure was analyzed by real time qPCR using CFX96 real-time PCR detection system (Biorad) and with iQ™ SYBR^®^ Green Supermix (Biorad). The protocol used for qPCR was: 95 °C for 5 min and 40 cycles at 95 °C for 10 s and 60 °C for 15 s followed by 60 °C for 5 s. Melting curves were performed at 60 °C for 5 s followed by gradual heating (0.5 °C/s) to 95 °C. Gene expressions were normalised to Ct values obtained for *Gallus gallus reference genes*: *Tbp*, *ActB*, G10, *Hmbs* and *Gapdh* using the formula: 2^−(Ct *Gallus gallus specific gene* − Ct *mean of Gallus gallus* reference genes)^. The primer sequences used in this study are listed in Table 1. To obtain a global view of gene expression in non-infected organoids, a heatmap analysis was performed using the R package Pheatmap [18]. Median of gene expression values were normalized with a Z-score approach and scaled by row (genes). When organoids were infected, a ratio between 2^−∆Ct^ of infected samples over non-infected samples was calculated and referred as fold change.

**Table 1:** **Primers used in this study.**

Gene name	Forward	Reverse
*Actb*	CCAGACATCAGGGTGTGATGG	CTCCATATCATCCCAGTTGGTGA
*Agr2*	CGCAGACGTATGAGGAAGCC	GGTTTCGTACACAAGGTTCAGG
*Bmi1*	CTGCTCAACATCAGGTCAGATA	TCTTCGTCAGCCACTTCTCCC
*Ccl20*	GGCACAAAGCAACCAAGATT	GGATTTACGCAGGCTTTCAG
*CD3d*	GACGCTCCCACCATATCAGG	ATCATTCCGCTCACCAAGGG
*CD8a*	CGAGGGGTCAAAGCAAG	TGTGGCTGGGAAGAAGG
*CD24*	AGCAAGTTCCACTTTGCCAGC	TTTCCATGTCCATGAGCGGTG
*CD44*	ACGAGGAGCAAAGCATGTGA	GTGAGCCGTCCTCATTGTCA
*Chga*	ATCTCCCTTCCTGTGACAAATG	GATCGGCAGTGGGTCTGGCTT
*Cldn 1*	AGATCCAGTGCAAGGTGTACG	CTGACAGACCTGCAATGATGAAG
*Cldn 10*	TCCAACTGCAAGGACTTCCC	GCACAGCCCACACAGTATGA
*CSF1R*	AGCTCTCACCTGGAACCAAC	AGGCTTCTCTTGTCCTTCAACC
*Cxcl8*	GCTCTGTCGCAAGGTAGGAC	GGCCATAAGTGCCTTTACGA
*CX3CR1*	GGCTGTCTCGGACCTTCTTT	TTGCAGGGGATAGTGCCAAG
*CyclinA2*	CAAGCTCCAGAATGAAACTC	GATGTAGACGAACTCTGCTA
*CyclinB2*	GTTCTGTCTCCTGTCCCTAT	AGCTCAAGCTGTCTCAGATA
*Fabp2*	GCTGACGGGACTGAACTTTCA	CGCTGTGAGTACTTTTCCATTATCTTT
*Fabp6*	ACTATAGACAAGGAAGCAGACATGGA	TCCATTTTGACTGTTGCCTTGA
*FLT3*	TTTGGACAGGTTCTCGGCTC	CTCCCGCCTTGCTAATTCCA
*G10*	AACAGCCTCTGCATCCACAGT	TCAAGGAAGGGTACGCTGACA
*Gapdh*	CCACAACATACTCAGCACCTGC	GTCCTCTGGCAAAGTCCAAG
*Jam2*	AGACAGGAACAGGCAGTGCT	TCCAATCCCATTTGAGGCTA
*Klf4*	CAAAGCCCAAGAGGGGACGG	GCAAACTTCCATCCGCAGCCT
*Krt7*	TGACAAAGGGCGTCTGGAGG	GAGCACCACGAACTCATTCTC
*Lgr5*	GCTGGCTTCTCTTCGTTCTCT	ATGTAGCCCCGTCACAGGAAA
*Lrig1*	GCATCGTGCTGACTTCGCTG	TAGTCTCATCTGTGTTGGTGAC
*Mmp7*	TACACACCTGACCTACCCCGA	GGCTGAAAAGCATGAGCTAATG
*MRC1L-B*	CGTTCCGGTGGATAGATGGAAG	GGGAGCAATACTTGGACGAA
*Muc2*	TGGAGGCAAAGTGTCTGCTCT	TAATAGCATGGGCATTTGGAGAT
*Mx1*	GGGGAACCAGCCACAAGATA	TTAGTGAGGACCCCAAGCGT
*Myd88*	CGTGCCAAAGACTTCAGAGC	ACCATCCTCCGACACCTTCTT
*Nfkb1*	CGAACAGCAGATGGACCGTA	TTACCCACCAAGCTGTGAGC
*Occludin*	CCGTAACCCCGAGTTGGAT	ATTGAGGCGGTCGTTGATG
*Olmf4*	CGACAGACGTGACTCCTCCTG	GGTGTGCTGGCTGGTAGTCTT
*Reg4*	GGAGGCAGAGGCACAGTGTC	CTCAGCGTGGCTGCTTCCTT
*Socs1*	CACGCACTTCCGAACCTTTC	ACTTCAGCTTCTCATGGGCG
*Stat1*	AAGCAAACGTAATCTTCAGGATAAC	TTTCTCTCCTCTTTCAGACAGTTG
*Stat3*	AAGGGTGACCCAATTGTCCA	TGTTAAACTTCCGGGACCCCC
*Tbp*	AGCTCTGGGATAGTGCCACAG	ATAATAACAGCAGCAAAACGCTTG
*Tjp1*	ACCGAGAGATGCTGGTACTG	GCACAGCCTCATTCTCATGG
*Tjp2*	CATTGTTCGGGAGGATGCTG	AGCCAGCCAGTTTCCTAGTT
*Tjp3*	GGATACAGTGCGGCAGATTG	TGGTAGCAGTGAAGAGGTGG
*Tlr1*	TGAGCTTCATGACCAGCCGT	TGGTTGTTTTGTAGGTCCACT
*Tlr15*	AGCTGAACTGCTGCCACATTT	TTTCCTCTGTTCTTCTTTGTCTGAATC
*Tlr3*	GATCCATGGTGCAGGAAGTTT	CTGGCCAGTTCAAGATGCAG
*Tlr4*	ACCTCAATGCGATGCACTCT	AGTCCGTTCTGAAATGCCGT
*Tlr5*	TGTGTTGTGACCAGGCAGTT	AATCTTCAGGCCAACGCAGA
*Vil1*	GCAACTTGTGTCAGGGCTCACC	CCAGCACGTCCAGTGGGAAGGT
*Wdr43*	TCCCTATTCTAGCGGCTGCGT	GTTCACAACTGGCGTCCTCAC

### Bacterial strains and infection conditions

The bacterial strains used in this study are listed Table 2. Bacteria were routinely grown in Luria–Bertani (LB, Sigma Aldrich, Saint-Louis, MO, USA) broth with shaking at 150 rpm at 37 °C overnight. The day of infection, 2.10^8^ bacteria were added per well of 24-well plate for 90 min at 37 °C in a humidified atmosphere at 5% CO_2_. After 90 min, infected organoids were washed with warm DMEM/F12 and then re-incubated further 90 min with fresh DMEM/F12. The time zero of post-infection (pi) was set at the beginning of the infection.

**Table 2:** **Bacterial strains and plasmids used in this study.**

Strains	Relevant characteristic(s)	Source or reference
Strains MC1061	*E. coli* hsdR mcrB araD139 Δ(araABC-leu)7679 ΔlacX74 galU galK rpsL thi	[46]
STm	*S. enterica* subsp. *enterica* ser. Typhimurium 14028 wild-type strain	ATCC

### Gentamicin protection assay

To quantify the number of internalized bacteria, DMEM/12 was removed and L-WRN medium containing gentamicin (100 µg/mL) was added to infected organoids (at 3 hpi) for 1 h 30 at 37 °C. The efficiency of the gentamicin (Gibco Life Technologies, Paisley, UK) treatment to kill extracellular bacteria in Matrigel^TM^ has been verified at 4 h 30 pi (Additional file 1). Matrigel^TM^ drops without organoids were infected with bacteria and treated with gentamicin as described above. Next, Matrigel^TM^ drops containing or not organoids were washed with DMEM/12 at 37 °C and then lyzed in cold distilled H_2_O for 30 min on ice by osmotic shock [19, 20]. Finally, the number of internalized and surviving bacteria at 4 h 30 pi was determined by plating appropriate dilution on Tryptic Soy Agar (TSA, BD Difco^TM^, Le Pont de Claix, France) and counted after overnight growth at 37 °C. The number of internalized bacteria was calculated as the ratio of colony forming units of lysates and inoculum and then, expressed relative to 100 organoids. To determine the intracellular replication level of *Salmonella* in infected organoids, L-WRN medium containing gentamicin at 100 µg/mL was replaced by cell culture medium containing gentamicin at 10 µg/mL for the remaining incubation time. The number of intracellular bacteria released from lyzed organoids at 24 hpi was numbered as at 4 h 30 pi. The ratio of the number of internalized bacteria at 24 hpi and 4 h 30 pi allowed to calculate the bacterial replication rate.

### Viability assay

The uninfected and infected organoids went through the same steps to assess the cell viability at 24 hpi using CellTiter-Glo 3D Cell Viability Assay according to the manufacturer’s recommendations (Promega, Madison, Wisconsin).

### Statistical analysis

The statistical differences between two groups were analyzed using a non-parametric Mann–Whitney test. *p*-values of 0.05 or less were statistically considered significant. Differences among three or more groups were analyzed using a Kruskall-Wallis test with a Dunn’s multiple comparisons post-test ANOVA (Prism, version 6.0; GraphPad Software, La Jolla, CA, USA).

## Results

### Organoids derived from chicken ileal and caecal crypts

**Figure 1:**
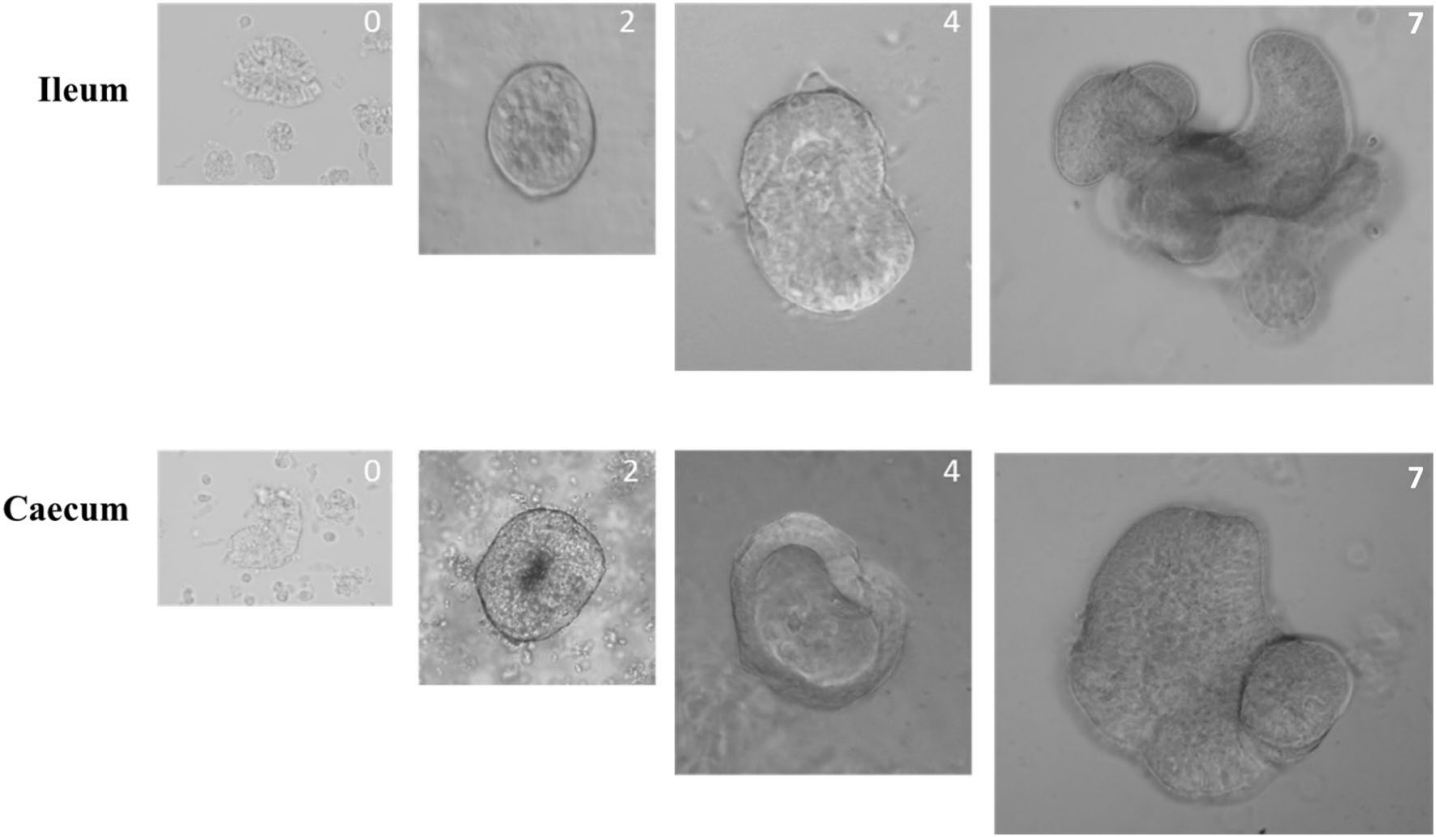
**Intestinal crypt cultures derived from chicken.** The images are representative of purified crypts derived from ileal and cecal segments cultured in Matrigel™ and L-WRN complete medium as described in Materials and Methods. Original magnifications of the crypt growth from day 0 to 7: day 0, × 40; day 2, × 20; day 4, × 10; day 7 × 4.

In chicks, the small and large intestine as well as the caecum, are colonized by *S.* Typhimurium. However, the caecum is the most heavily colonized intestinal segment [4]. To compare in vitro the direct interaction of *S.* Typhimurium with chicken ileal and caecal epithelial cells, we attempted to design organoid culture derived from intestinal crypts of the two segments. Intestinal organoids were chosen as they recapitulate the cellular complexity and architecture of the intestinal epithelium in vitro without variability of in vivo experiments introduced by immune cells or the intestinal microbiota [21]. Purified ileal and caecal crypts were resuspended in Matrigel™ to form structures in 3D and cultured in L-WRN complete medium which has been described to allow obtention of intestinal epithelial lineages [21]. Figure 1 illustrates the growth of crypts to form mature organoids. By 2 days of culture, the crypt rapidly became sealed and had a rounded shape (Figure 1). This structure becomes larger over time and starts budding, showing an enclosed central lumen (Figure 1, day 4). By 7 days of culture, numerous buds are observed, demonstrating a typical morphology of crypt-like structure and characteristic of a mature organoid (Figure 1) [22]. Organoids derived from ileum are generally composed of bigger buds compared to organoids derived from caecum (Figure 1). Few days after crypt seeding, the cells are exfoliated into the organoid lumen, leading to an accumulation of apoptotic cells visualized by a dark lumen (Additional file 2). Organoids were enzymatically dissociated weekly and replaced in Matrigel™ to form new organoids. They were kept for at least 7 passages (data not shown).

### Characterization of chicken intestinal organoid

**Figure 2:**
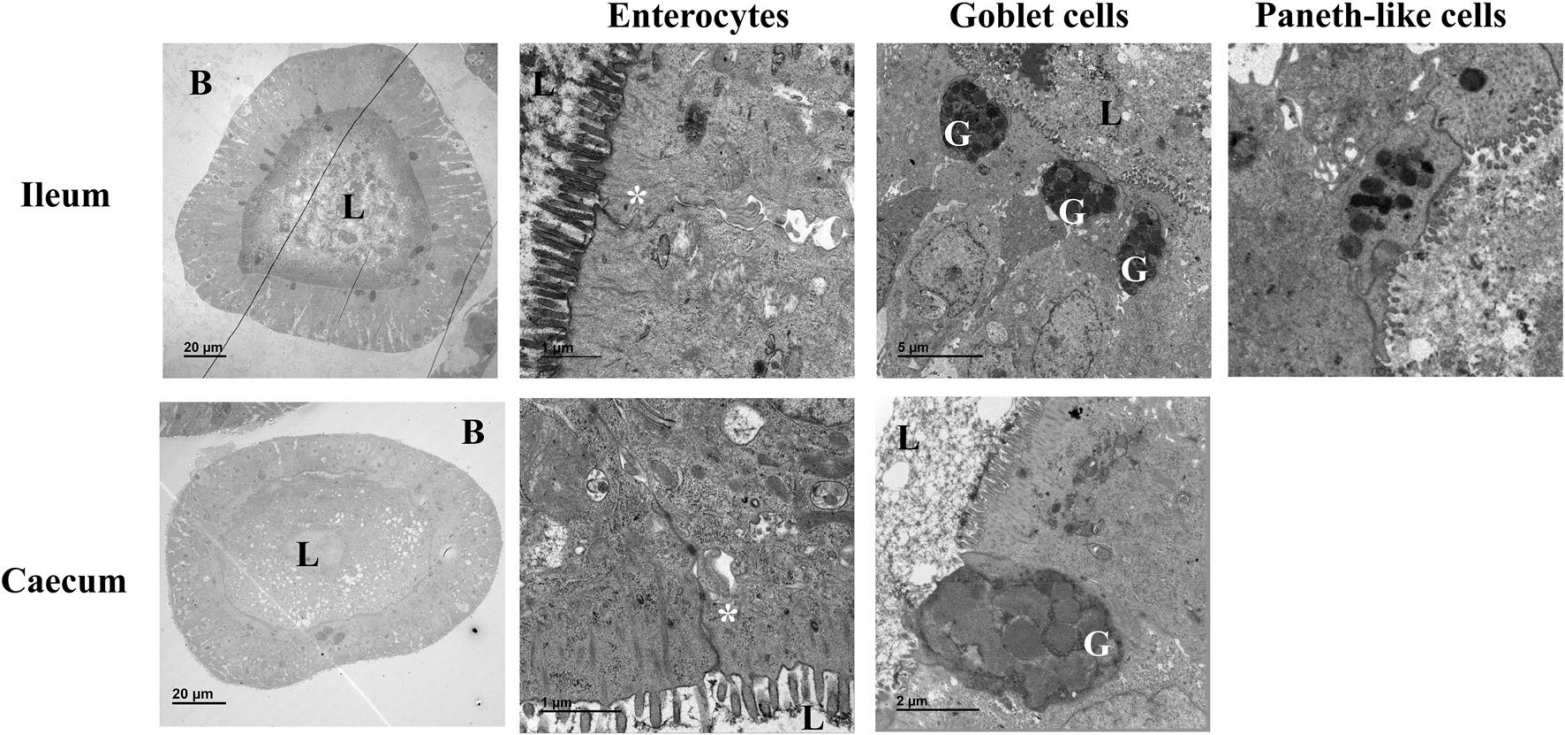
**Morphological analysis.** TEM images of ileal and cecal organoids derived from chicken after 7 days of culture in Matrigel™ with L-WRN complete medium as described in Materials and Methods. The scale bars are indicated. G, goblet cells, *cell-to-cell interaction. B, basal lamina; L, lumen.

**Figure 3:**
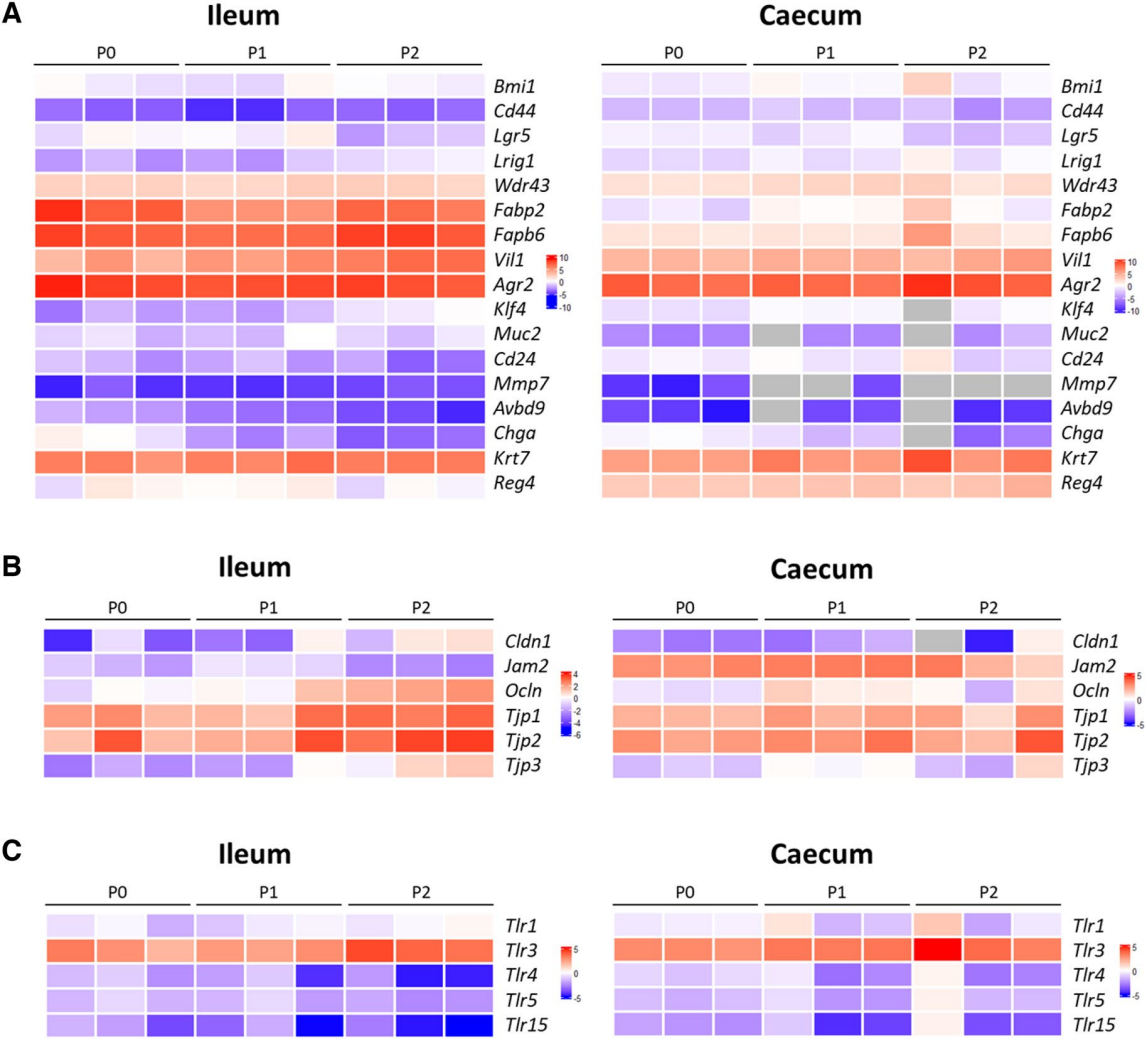
**Relative gene expression of cell and function markers in chicken ileal and caecal organoids.** Gene expression in organoids was analyzed by qRT-PCR. Heat maps show the Log_2_^−∆Ct^ of a range of epithelial cell genes and colours represent scaled values of gene expression with blue for low and red for high values. P: organoid passage number (3 samples by passage). **A** Cell type genes **B** Tight-Junction genes **C** TLR genes.

To be as close as possible to the in vivo intestine with the diversity of epithelial cells displaying specific functions, organoids must harbor intestinal stem cells, progenitor cells and differentiated cells, representing the intestinal epithelium and recapitulating the epithelial functions. Ultrastructural analyses of ileal and caecal organoids derived from chicken at day 7 revealed highly polarized organization of enterocytes with their typical apical brush border and cell-to-cell interactions such as adherent junctions (Figure 2). Mature ileal and caecal organoids derived from chicken thus have basal-out conformation with the apical side facing the lumen of the organoid, containing shed apoptotic cells, that occur during intestinal renewal (Figure 2). Moreover, we clearly distinguished the presence of goblet cells with intracellular mucin granules (Figure 2). The presence of rare cells containing dense cytoplasmic vesicles could be detected in ileal organoids but not in caecal organoids that could be indicative of Paneth-like cells (Figure 2). To further characterize the cellular composition of ileal and caecal organoids, the expression of some genes, characteristic of intestinal cell type and barrier markers was assessed by qRT-PCR in ileal and caecal chicken organoids by 7 days of culture of various passages P0, P1 and P2. Our data shown in Figure 3A suggest that ileal and caecal chicken organoids recapitulate the cellular diversity of the intestinal epithelium. *Bmi1*, *CD44*, *Lgr5*, and *Wdr43* genes revealed the presence of stem and transit-amplifying cells and *Lrig1* and *CD24* genes, proliferation regulators. Enterocytes can be identified by the expression of *Fabp2*, *Fabp6* and *Vil1*. *Agr2*, *Klf4*, *Muc2* gene expression is currently used to detect goblet cells. *CD24* and *Mmp7* gene expression suggest the presence of Paneth-like cells and *Chga*, *Krt7*, *Reg4* of enteroendocrine cells. In chicken intestine, *AvBD9* was detected in enteroendocrine cells [23], this gene expression could be associated to this cell type. Expression of several genes encoding components of tight junctions *Cldn1*, *Jam2*, *Ocln* and *Tjp1*,* 2*,* 3* was detected in the organoids (Figure 3B), consistent with the detection of these structures by TEM (Figure 2). Some TLRs are also expressed in ileal and caecal organoids suggesting a potential response of organoids to PAMP stimulation or infection (Figure 3C).

Nash et al. as well as Orr et al. described the presence of immune cells in chicken enteroid cultures derived from ED18 embryonic chickens, potentially isolated during the crypt purification process [14, 15]. The presence of T cells, dendritic cells and macrophages was searched by analyzing gene expression of specific marker in ileal and caecal chicken organoids. *CD3d* and *CD8α* were used for T cell, *FLT3* and *CX3CR1* for dendritic cell and *CSF1R*, *MRC1* for macrophages and *Vil1* for enterocytes as control. The transcriptional analysis showed only detection of *CD8α* expression as well as *FLT3* and *CX3CR1* but in a lesser number of samples (Additional file 3) in both ileal and caecal organoids, suggesting the presence of immune cells in our 3D chicken intestinal organoid model. However, as the presence of immune cells could not be observed by TEM, it suggests a very weak proportion of these cells compared to epithelial cells.

Taken together, our data demonstrated that in chicken organoids cultivated in Matrigel™ and L-WRN complete medium, the intestinal hierarchy derived from stem cell is maintained as well as the epithelial barrier function. As the intestinal physiology is closely reproduced in ileal and caecal organoids derived from chicken during passages, these models were therefore used to study the intracellular *S.* Typhimurium replication in the chicken epithelium and the cell responses.

### *Salmonella* infection in chicken ileal and caecal organoids

**Figure 4:**
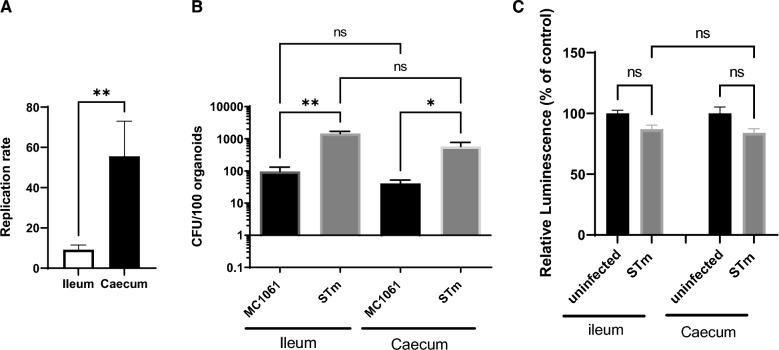
**Interaction of *S*. Typhimurium with the chicken intestinal epithelium.** Organoids were infected with either MC1061 (MC1061) or *S.* Typhimurium (STm). Strains were grown in LB medium and then deposited onto organoids before gentamicin treatment. **A **Replication of *S.* Typhimurium in chicken intestinal organoids. The bacterial replication rate was determined at 24 hpi and the mean ± SEM obtained from at least two independent experiments with 4 infected wells per experimental condition are represented. **B** Invasiveness of *S.* Typhimurium in chicken organoids. The number of internalized bacteria was determined at 4 h 30 pi. Results are mean ± SEM obtained from at least two independent experiments with 4 infected wells per experimental condition. **C** Cell viability of uninfected and infected chicken organoids. At 24 hpi, the relative luminescence as indicator of viability was generated using Cell Titer-Glo 3D assay (**p-value < 0.01, *p-value < 0.05, ns: non-significant).

The intracellular survival of *S.* Typhimurium in ileal and caecal organoids were compared by performing a gentamicin protection assay at 4 h 30 pi and 24 hpi. To do that, the ileal and caecal 3D organoids were first incubated with the bacteria for 90 min to allow bacteria to cross the Matrigel™ and to contact the basal cell surface of the organoids. Then, the extracellular medium containing bacteria was replaced with fresh DMEM medium and incubated again 90 min. Next, the infected organoids were incubated with L-WRN complete medium containing 100 µg/mL gentamicin for 90 min and the number of internalized bacteria was quantified (4 h 30 pi time point). To quantify the number of internalized bacteria at 24 hpi, the ileal and caecal 3D organoids were incubated at 4 h 30 pi with L-WRN complete medium containing 10 µg/mL gentamicin overnight (24 hpi time point). The bacterial replication rate was calculated by dividing the number of internalized bacteria at 24 hpi by the one obtained at 4 h 30 pi. As shown in Figure 4A, *S.* Typhimurium is able to replicate and survive in both ileal and caecal organoids. However, the *S.* Typhimurium rate is about 8 in ileal organoids, while it is about 53 in caecal organoids, when the number of internalized bacteria is similar at 4 h 30 pi (Figure 4B). Of note, the comparison of cell viability between ileal and caecal organoids at 24 hpi revealed that there is no significant difference (Figure 4C). A non-invasive *E. coli* strain (MC1061) was used as control for the quantification of internalized bacteria to demonstrate that the internalization process observed does not result from intrinsic ability to ingest bacteria independent of any virulence factors. As shown in Figure 4B, the number of internalized *E. coli* strain is negligible (significantly lower, by 94.06% ± 5.5 in ileal organoids and 93.16% ± 5.7 in caecal organoids, compared to *S.* Typhimurium set at 100%), leading to the conclusion that the internalization process observed in chicken organoids is specific of *Salmonella*.

Taken together, these data demonstrated that the uncomplicated infection model of basal-out chicken organoid recreate an intracellular environment allowing *Salmonella* replication, a common crucial step to colonize the epithelium. This model was therefore used for the remaining experiments.

### Modulation of the intestinal proliferation by *Salmonella*

**Figure 5:**
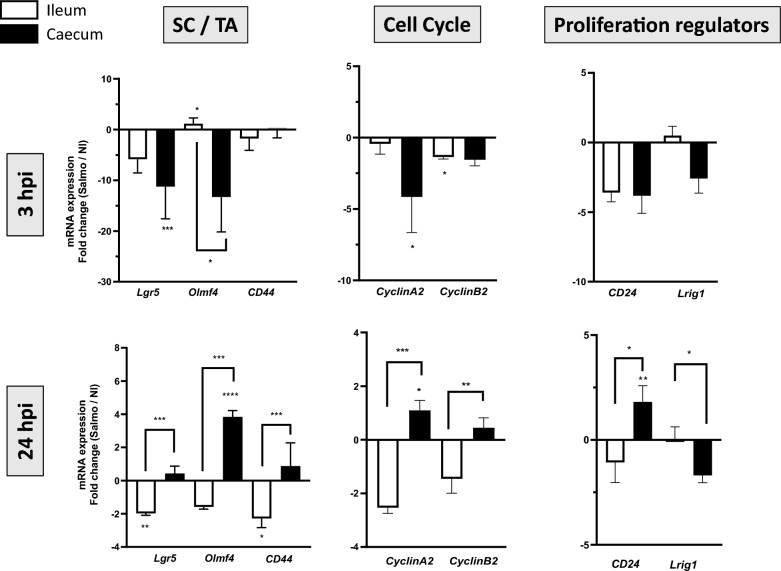
**Impact of *S.* Typhimurium on intestinal cell proliferation.** The relative gene expression of specific genes involved in intestinal cell proliferation (*Lgr5*, *Olmf4*, *CD44*, *CyclinA2*, *B2*, *CD24* and *Lrig1*) was analyzed by qRT-PCR in infected and non-infected organoids of caecum (black boxes) and ileum (white boxes). The values of gene expression were calculated with 2^−∆Ct^ for each sample. Results on histograms correspond to the mean ± SEM of the ratio of 2^−∆Ct^ (fold change) between infected and non-infected conditions and obtained from 2 independent experiments with *n* = 4–6 samples per experiment. A first statistical analysis was performed to compare 2^−∆Ct^ values of infected and non-infected (Mann–Whitney test, ***p < 0.001, **p < 0.01, *p < 0.05 on the top of boxes). The fold-change values of caecal organoids (Infected/non-infected) and ileal organoids were statistically compared (Mann–Whitney test, ^###^*p* < 0.001, ^##^*p* < 0.01, ^#^*p* < 0.05)

As the interaction of *S.* Typhimurium with chicken intestinal epithelial cells leads to a reduced proliferation of the intestinal stem cell [24], we next investigated the impact of *S.* Typhimurium replication on the intestinal cell proliferation in ileal and caecal organoids. To do that, the mRNA expression level of specific genes involved in intestinal cell proliferation was analyzed with 3D ileal and caecal organoids uninfected or infected with *S*. Typhimurium at 3 h and 24 hpi. As control, non-infected cells were used. Gene expression results are presented as fold-changes over the non-infected cells. We did not choose to use the *E. coli* strain even without internalization capacity, as *E. coli* expresses some factors that can lead to host cell responses such as those that can stimulate TLR responses. *Lgr5*, *Olmf4* and *CD44* were used as marker of stem/ transit-amplifying cells (SC/TA), *CyclinA2* and *B2* for the cell cycle and the proliferation regulators *CD24* and *Lrig1*, as negative regulator of EGFR signaling. As shown Figure 5, despite similar invasiness at 3 hpi of *S.* Typhimurium in ileal and caecal organoids (Figure 4A), *S.* Typhimurium repressed expression of stem/ transit-amplifying cell, cell cycle and proliferation regulators related genes but to a lesser extend in ileal organoids at 3 hpi. At 24 hpi, when a higher intracellular replication rate of *S.* Typhimurium is observed in caecal organoids, expression of stem/ transit-amplifying cells (SC/TA), cell cycle and proliferation regulators related genes was less modulated than in ileal organoid. Taken together, these data suggest that the cells of the *S.* Typhimurium infected caecal organoids are more proliferative compared to those of ileal organoids.

### Modulation of the intestinal responses by *Salmonella*

**Figure 6:**
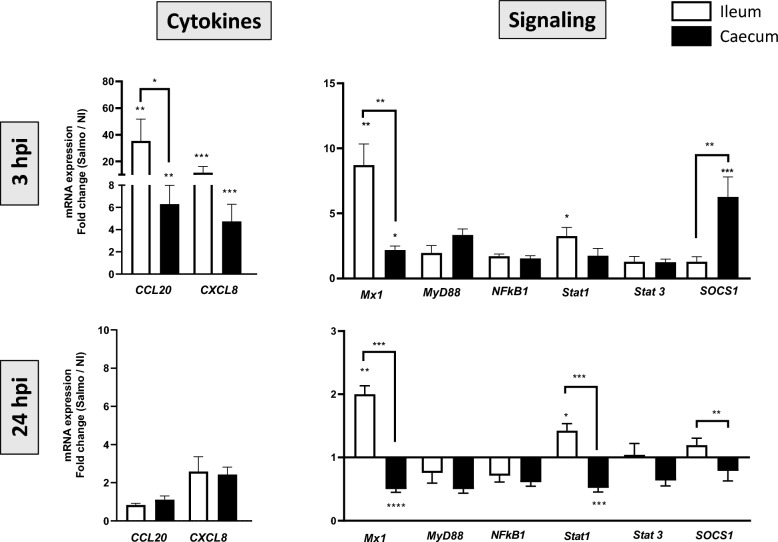
**Impact of *S.* Typhimurium on immune host responses.** The relative gene expression of specific genes involved in host response was analyzed by qRT-PCR in infected and non-infected organoids of caecum (black boxes) and ileum (white boxes). The values of gene expression were calculated with 2^−∆Ct^ for each sample. Results on histograms correspond to the mean ± SEM of the ratio of 2^−∆Ct^ (fold change) between infected and non-infected conditions and obtained from 2 independent experiments with *n* = 4 to 6 samples per experiment. A first statistical analysis was performed to compare 2^−∆Ct^ values of infected and non-infected (Mann–Whitney test, ***p < 0.001, **p < 0.01, *p < 0.05 on the top of boxes). The fold-change values of caecal organoids (Infected/non-infected) and ileal organoids were statistically compared (Mann–Whitney test, ^###^*p* < 0.001, ^##^*p* < 0.01, ^#^*p* < 0.05)

*S.* Typhimurium stimulates specific host receptors triggering a variety of generic responses directed at controlling pathogen spread [25]. In this study, to better characterize the impact of *Salmonella* replication on intestinal cell response, we analyzed the mRNA responses of CCL20 and CXCL8, two well described cytokine and chemokine produced by infected epithelial cell lines in vitro [26, 27] and measured in vivo in chicken epithelial cells [28, 29]. We observed a significant increased mRNA expression of CCL20 and CXCL8 as soon as 3 hpi which is significantly higher in ileal organoids compared to caecal organoids (Figure 6). We also explored the response of signaling molecules involved in host cell immune response. As soon as 3 hpi, we observed a significant increase of *Mx1, Stat1* and *Socs1* mRNA expression in infected organoids compared to uninfected organoids (Figure 6). As observed above for chemokine response, *Mx1* and *Stat1* increase are higher in ileal organoids compared to caecal organoids, whereas *Socs1*, as a negative feedback regulator of cytokines, is higher in caecal organoids (Figure 6).

Compared to non-infected organoids, the Mx1 and Stat1 responses were still significantly increased at 24 hpi in infected ileal organoids but in a lesser extent, whereas they are significantly decreased in caecal organoids.

## Discussion

The gastrointestinal tract of chicken harbors differences when compared to the mammals one. It displays a shorter size relative to body length, and the size and role of the caeca are clearly different [30, 31]. Moreover, at the cellular composition level, it was reported that the proliferation of epithelial cells in the small intestine of the chicken is not restricted to crypts (80%), and is also present along the villus. This was demonstrated by measuring the uptake rate of 3H-thymidine, immunostaining of 5-bromo-2-deoxyuridine (BrdU) and proliferating cell nuclear antigen (PCNA) on chicken small epithelium [32, 33]. This discrepancy was recently described on chicken intestinal organoids [14].

*S.* Typhimurium can colonize the different parts of the intestinal tract in chicks older than 3 days of age for several weeks [34] and the caecum is the segment most highly colonized in infected chicks [4, 5]. However, the impact of the intracellular fate of *S.* Typhimurium allowing the chicken intestinal colonization was limited due to the absence of in vitro intestinal epithelium model.

In this study, we cultured chicken intestinal organoids obtained by seeding ileal and caecal crypts in Matrigel™ with L-WRN complete medium. These organoids contain the different cell types, forming the intestinal epithelium and some immune cells, according to visualization by TEM and gene expression of specific cell markers as previously described [14, 15]. Moreover, epithelial functionalities are reproduced in organoid model as key components of the epithelial barrier (e.g. tight junction proteins) are found expressed. In addition to the initial characterization by Zhao et al. [11], we demonstrated that there is no difference in TLR expression between ileal and caecal organoids (Figure 3). By transmission electronic microscopy, microvilli at the luminal side are observed, indicating that epithelial cells were polarized in our intestinal organoids derived from chicken (Figure 2). However, in this 3D model, the apical side of organoid epithelial cells, which is the first target of *S.* Typhimurium, is not directly accessible and this leads to a basolateral exposure of *S*. Typhimurium when directly added in the medium. Other approaches have been described to mimic intestinal infections and circumvent this difficulty to access apical side of epithelial cells. It is possible to inject *S*. Typhimurium into organoid lumen as described in human intestinal organoids for understanding the initial steps of *Salmonella* pathogenesis [35, 36]. However, this method needing specific technical skills, is relatively labor intensive and concerns have been raised regarding unintended leak into the medium and reproducibility due to variable volume injected in each organoid [21]. A recent study showed that chicken enteroid cell polarity can be reversed by removal of the Matrigel™ [15]. *S.* Typhimurium infection was performed in these conditions but only for 8 h and no cell response was investigated. The main drawback of these apical-out organoids is that they originated from cultivated crypts and no passage is possible for a long term culture. This also can explain the presence of leukocytes in the culture. Moreover, it is difficult to be sure that the polarity of all organoids is reversed leading to potential variability in these culture conditions. Another method to facilitate the access to the apical side is the culture of 2D monolayers of organoid epithelial cells [14]. No infection by *Salmonella* was performed in this model but a stimulation with *Salmonella* LPS revealed the possible induction of an innate immune response with IL-6 and IL-8 increased expression 6 h after stimulation.

*S.* Typhimurium has the ability to adhere to and invade intestinal epithelium at both apical and basolateral side but once internalized, *Salmonella* intracellularly replicate independently of the internalization mechanism and its impact have never been investigated. To invade non phagocytic cells, *S.* Typhimurium expresses three known internalization factors (T3SS-1, Rck and PagN) [2]. But it is still unclear when *S.* Typhimurium expresses and uses them in the intestine. Expression of the T3SS-1 and the secretion of the T3SS-1 effectors are influenced by various environmental signals such as oxygen concentration and bacterial growth. Thus, the culture conditions affect *S.* Typhimurium invasion efficiency [37, 38]. In this study, *S.* Typhimurium overnight grown cultures were used, which does not allow an optimal expression of the T3SS-1 as well as T3SS-1 dependent invasion. The impact of the T3SS-1 in this study is therefore limited.

The lack of avian chicken cell lines leads to very little knowledge about the intracellular fate of *S.* Typhimurium, a crucial step allowing the chicken intestinal epithelium colonization. Using the basal-out organoid model derived from ileal and caecal segment, we showed that *S.* Typhimurium replicates 6 times more in caecal than in ileal epithelium. Our findings suggest that the uncomplicated infection model of basal-out chicken organoid recreates an intracellular environment allowing *Salmonella* replication, a common crucial step to colonize the epithelium. This infection model is therefore appropriate to investigate the impact of intracellular *Salmonella* on avian intestinal cell response.

In our study at 24 hpi, a higher quantity of internalized *S.* Typhimurium was observed in chicken organoids derived from caecum, while the invasiveness in ileal and caecal organoids was similar. This could reflect a different apoptosis induction in infected organoids. To eliminate this hypothesis, the cell viability in uninfected and infected chicken organoids derived from ileum and caecum has been investigated. However, no significant difference was observed between infected organoids derived from ileum and caecum (Figure 4C). It thus appears that the higher number of internalized bacteria at 24 hpi was linked to a better bacterial replication and not to apoptosis induction in ileal organoids. A tendency (but non-significant) decrease of cell viability was observed in infected organoids compared to non-infected organoids (Figure 4C), which is in line with previous study in cell lines. In fact, following infection with *Salmonella*, human colon epithelial cells are shown to undergo apoptosis, requiring bacterial entry and replication. The ensuing phenotypic expression of apoptosis occurs 12–18 h after bacterial entry [39].

In cell culture and mouse model, the Wnt/β catenin signaling pathway that is known to regulate stem cells seems to be suppressed after infection with *S.* Typhimurium [40]. Similar results are observed in chicken model infected with *S.* Typhimurium. Zhang et al. demonstrated in small intestine of chicken that *S.* Typhimurium infection caused a decrease in crypt depth, as well as a reduction in the number of proliferative cells per crypt [24]. In this study, we observed that *S.* Typhimurium infection causes a decrease in mRNA expression of genes specific to stem/ transit-amplifying cells, cell cycle, proliferation regulators and EGFR signaling, thus demonstrating a negative modulation of intestinal cell proliferation. Our results are in line with the results obtained in vivo, validating intestinal basal-out organoid as in vitro model to modelling *Salmonella* infection. However, a lower impact of *S.* Typhimurium on proliferation of intestinal stem/ transit-amplifying cells is observed in infected caecal organoids than in ileal organoids. As Zhao et al*.* have highlighted that *S.* Typhimurium replication is greatly boosted when host cells were in G2/M phase [41], this allows us to emit the hypothesis that *S.* Typhimurium preferentially replicates in a proliferative environment. This hypothesis is currently under verification in our laboratory.

The intestinal epithelium is the first line of defense or communication with intraluminal bacteria. A number of bacteria alters pro-inflammatory cytokine and chemokine production by the gut epithelium and particularly CXCL8 and CCL20 which are rapidly induced at the transcriptional level. *S.* Typhimurium induces CXCL8 transcription and secretion by increasing intracellular [Ca2 +]. In chicken intestinal organoids, we also observed a rapid IL-8 and CCL20 response after *S.* Typhimurium infection compared to non-infected organoids. These increased expressions after infection were significantly higher in ileal organoids compared to caecal organoids despite similar invasion rate at 3 hpi. Enhanced expressions of CXCL8 and CCL20 after infection were consistent with prior in vivo studies in chickens, and in mouse and human organoid models [27, 29, 42], thus establishing the chicken organoids as a valuable infection model for *Salmonella*. CXCL8 and CCL20 responses were shown to be independent on TLR expression [43] and on the ability of *S.* Typhimurium to invade epithelial cells [44, 45]. We can therefore hypothesize that the CXCL8 and CCL20 differential responses after infection could be attributed to a differential intracellular fate of *Salmonella* in ileal and caecal chicken epithelial cells. Moreover, in addition to stimulation of pro-inflammatory gene expression, *S.* Typhimurium also induced the expression of several genes whose products limit the immune response, such as the suppressors of cytokine signaling (SOCS) and members of the DUSP family of tyrosine phosphatases (DUSP1, DUSP2, DUSP4, DUSP5, DUSP6, DUSP8) in a human epithelial cell line [43]). The increased SOCS1 response observed after infection of chicken organoids still confirms the relevance of the model.

Taken together, this study shows that the intestinal organoid model is suited to mimic the in vivo *Salmonella* infection, making them promising in vitro model to specifically decipher the interactions of *Salmonella* with the intestinal epithelium. Moreover, it illustrates the importance of the gut segment used to purify stem cells and derive organoids. In addition, the organoid model carries a great potential to drastically reduce the number of animals used in the future. Thus, we believe that the use of the intestinal organoid model derived from different mammalian species such as human, mouse and chicken would contribute to better characterize the interaction of *S.* Typhimurium with intestinal epithelium according to the host and to establish a link with its potential role in the pathogenicity of *Salmonella*.

## Supplementary Information

**Additional file 1. Efficiency of the gentamicin treatment.** Matrigel™ drop in 24 wells plate containing or not organoids were cultured with L-WRN complete medium. Matrigel™ drops were infected with *S.* Typhimurium before gentamicin treatment and the number of colony forming units was determined at 4 h 30 pi as described in Materials and methods. Results are mean ± SEM obtained from two independent experiments with at least 3 infected wells per experimental condition.

**Additional file 2. Examples of mature organoid cultures containing dead cells in the organoid central area.** Organoids were derived from chicken ileum and caecum. 4 × magnification. Arrows show the accumulation of dead cells in lumen.

**Additional file 3. Detection of immune cells by qRT-PCR.**  The relative gene expression of specific marker of immune cells was analyzed by qRT-PCR in chicken organoids derived from ileum and caecum. The values of gene expression were calculated with 2-ΔCt for each sample. Each point on the graph represented detected positive samples.

## Data Availability

Not applicable.
