# Supplementary figures and images for: Differential Salmonella Typhimurium intracellular replication and host cell responses in caecal and ileal organoids derived from chicken
Source: Vet Res. 2023 Jul 31;54:63. doi: 10.1186/s13567-023-01189-3 (PMC10391861; doi:10.1186/s13567-023-01189-3)

## Slide 1
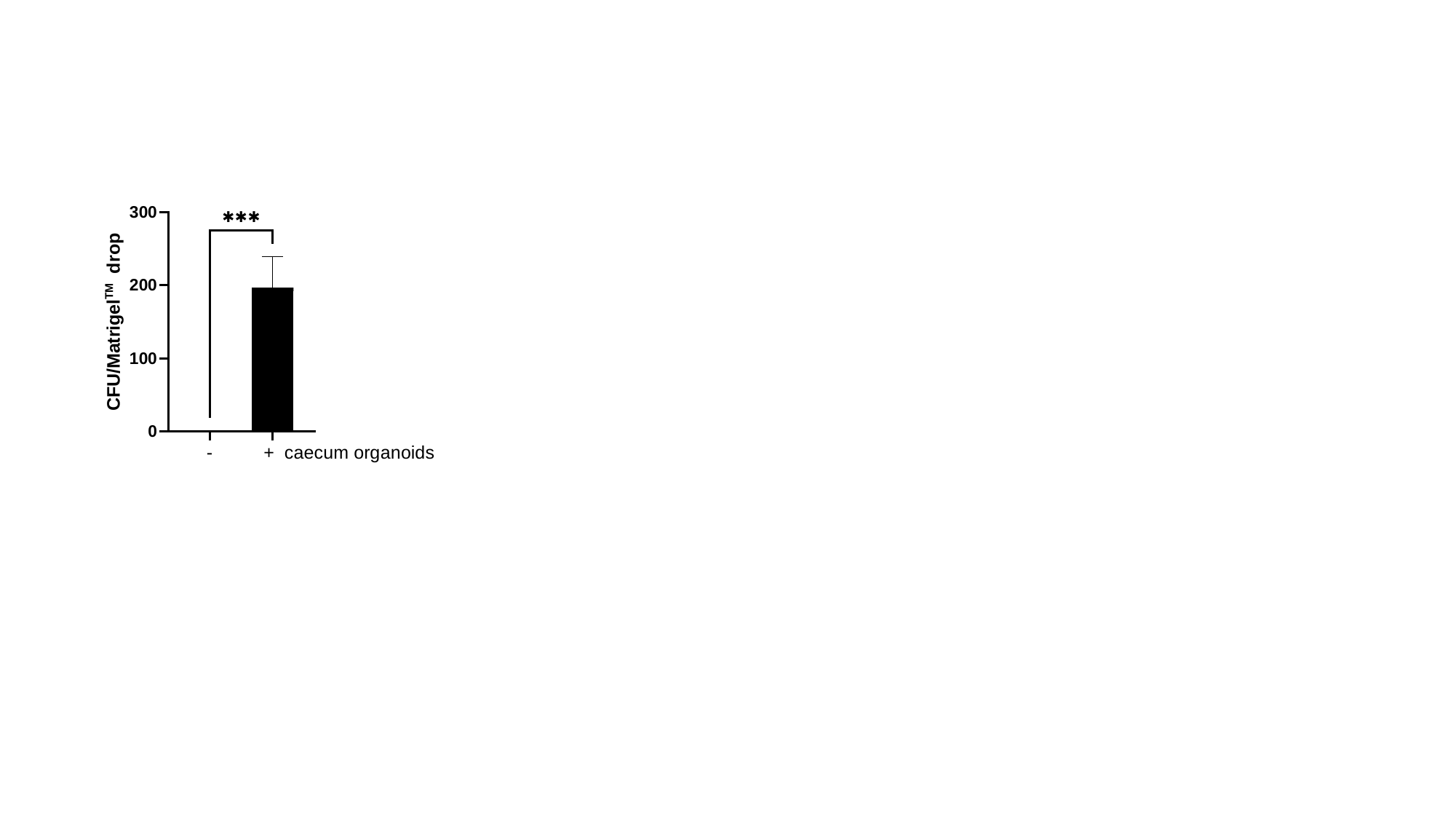

Supplement: Supplementary file 1 — Additional file 1. Efficiency of the gentamicin treatment. Matrigel™ drop in 24 wells plate containing or not organoids were cultured with L-WRN complete medium. Matrigel™ drops were infected with S. Typhimurium before gentamicin treatment and the number of colony forming units was determined at 4 h 30 pi as described in Materials and methods. Results are mean ± SEM obtained from two independent experiments with at least 3 infected wells per experimental condition. [file 13567_2023_1189_MOESM1_ESM.pptx]

## Slide 1
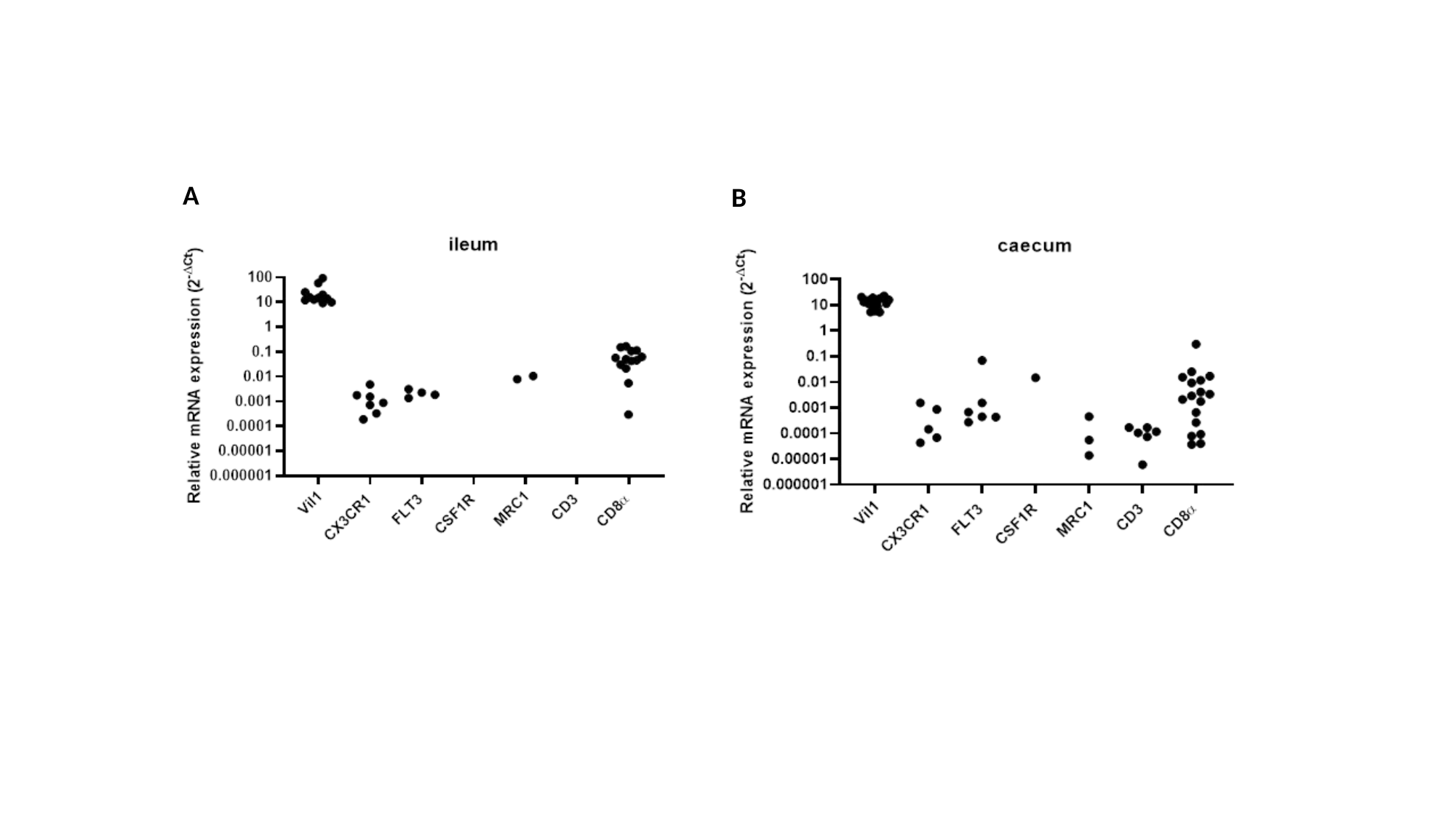

A
B

Supplement: Supplementary file 2 — Additional file 2. Examples of mature organoid cultures containing dead cells in the organoid central area. Organoids were derived from chicken ileum and caecum. 4 × magnification. Arrows show the accumulation of dead cells in lumen. [file 13567_2023_1189_MOESM2_ESM.pptx]

## Slide 1
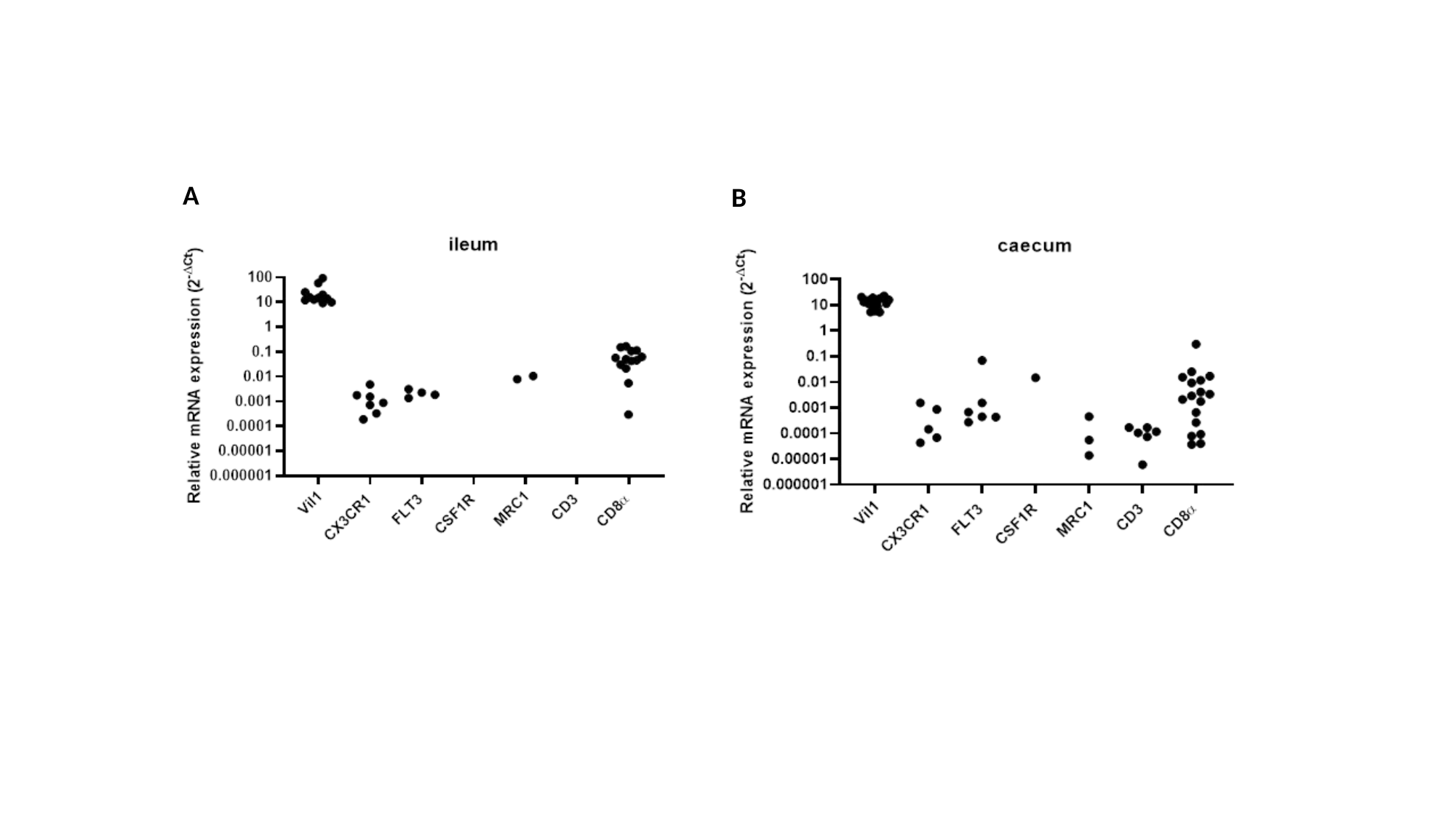

A
B

Supplement: Supplementary file 3 — Additional file 3. Detection of immune cells by qRT-PCR. The relative gene expression of specific marker of immune cells was analyzed by qRT-PCR in chicken organoids derived from ileum and caecum. The values of gene expression were calculated with 2-ΔCt for each sample. Each point on the graph represented detected positive samples. [file 13567_2023_1189_MOESM3_ESM.pptx]
